# *Begonia wui-senioris* (sect. *Platycentrum*, Begoniaceae), a new species from Myanmar

**DOI:** 10.1186/1999-3110-55-13

**Published:** 2014-02-01

**Authors:** Ching-I Peng, Hong Wang, Yoshiko Kono, Hsun-An Yang

**Affiliations:** 1 Herbarium (HAST), Biodiversity Research Center, Academia Sinica, Nangang, Taipei 115 Taiwan; 2 Xishuangbanna Tropical Botanical Garden, Chinese Academy of Sciences, Mengla, 666303 China

**Keywords:** *Begonia wui-senioris*, Begoniaceae, Chromosome number, Limestone, Myanmar, New species, Sect. *Platycentrum*

## Abstract

**Background:**

The flora of Myanmar is under-collected compared with all other tropical Asian countries. An unknown *Begonia* was grown from seeds collected from a limestone hill in Central Myanmar, and compared with potentially allied species.

**Results:**

The unknown *Begonia* is rhizomatous, has peltate leaves, 2-locular ovaries, and is evergreen. It is clearly assignable to sect. *Platycentrum*. Only two other species of *Begonia*, *B. josephii* and *B. subperfoliata*, in Myanmar have peltate leaves, but they are deciduous tuberous plants with 3-locular ovaries and belong to sect. *Diploclinium*.

**Conclusions:**

Thorough studies of literature and herbarium materials support the recognition of a new species, *Begonia wui-senioris*, which is fully described and illustrated. *Begonia wui-senioris* has the lowest chromosome number (2*n* = 14) for the genus.

**Electronic supplementary material:**

The online version of this article (doi:10.1186/1999-3110-55-13) contains supplementary material, which is available to authorized users.

## Background

The most recent checklist for the *Begonia* of Myanmar (Hughes, 2008) reports 57 species as native. Almost concurrently, a new species was reported, *Begonia hayamiana* (sect. *Sphenanthera*) (Tanaka and Hughes 2007), bringing to the total to 58. In 2009, Hong Wang, the 2nd author of this article collected seeds of a *Begonia* from Central Myanmar and sent them to the senior author, Peng, who germinated them and raised them to maturity in the experimental greenhouse of Academia Sinica, where they were carefully examined. Upon consulting herbarium materials from major herbaria, both domestic and abroad, the plants were found not to belong to any species known from Myanmar. However, a herbarium specimen in E, collected from the region where our seeds were gathered, corresponds to our living material. That specimen was annotated in 1992 as “*Begonia lacei* C. Y. Wu, sp. nov. ined.,” by the late Prof. Zhengyi Wu, renowned botanist of the Chinese Academy of Sciences. This unpublished taxon was excluded from the current checklist for Myanmar (Hughes 2008).

## Methods

### Materials

Living plants of *Begonia wui-senioris* were raised from seeds collected from ca. 200 km northeast of Mandalay, Myanmar and grown in an experimental greenhouse at Academia Sinica. The type collection (*Peng 22199*) was gathered from the greenhouse and deposited at HAST herbarium.

### Chromosome preparations

Root tips of *Begonia wui-senioris* were pretreated with 2 mM 8-hydroxyquinoline solution at 15-18°C for 6–8 h and then fixed overnight in ethanol-acetic acid (3:1) below 4°C. They were macerated in an enzyme mixture [2% Cellulase Onozuka R-10 (Yakult Honsha, Tokyo, Japan) and 1% Pectolyase (Sigma, St. Louis, MO, USA)] at 37°C for about 1 h. Chromosomes were stained in 2% Giemsa solution (Merck, Darmstadt, Germany). Classification of the chromosome complements, based on centromere position at mitotic metaphase, follows Levan et al. (1964).

### Cryo scanning electron microscopy

Fresh leaves of *Begonia wui-senioris* were dissected and attached to a stub. The samples were frozen with liquid nitrogen slush, then transferred to a sample preparation chamber at -160°C and etched for 15 min at -85°C. After etching, the temperature reached -130°C for sample fracturing and coating. After coating, the samples were transferred to the SEM chamber and observed at -160°C with a cryo scanning electron microscope (FEI Quanta 200 SEM/Quorum Cryo System PP2000TR FEI).

## Results and discussion

### Species description

**Figure 1:**
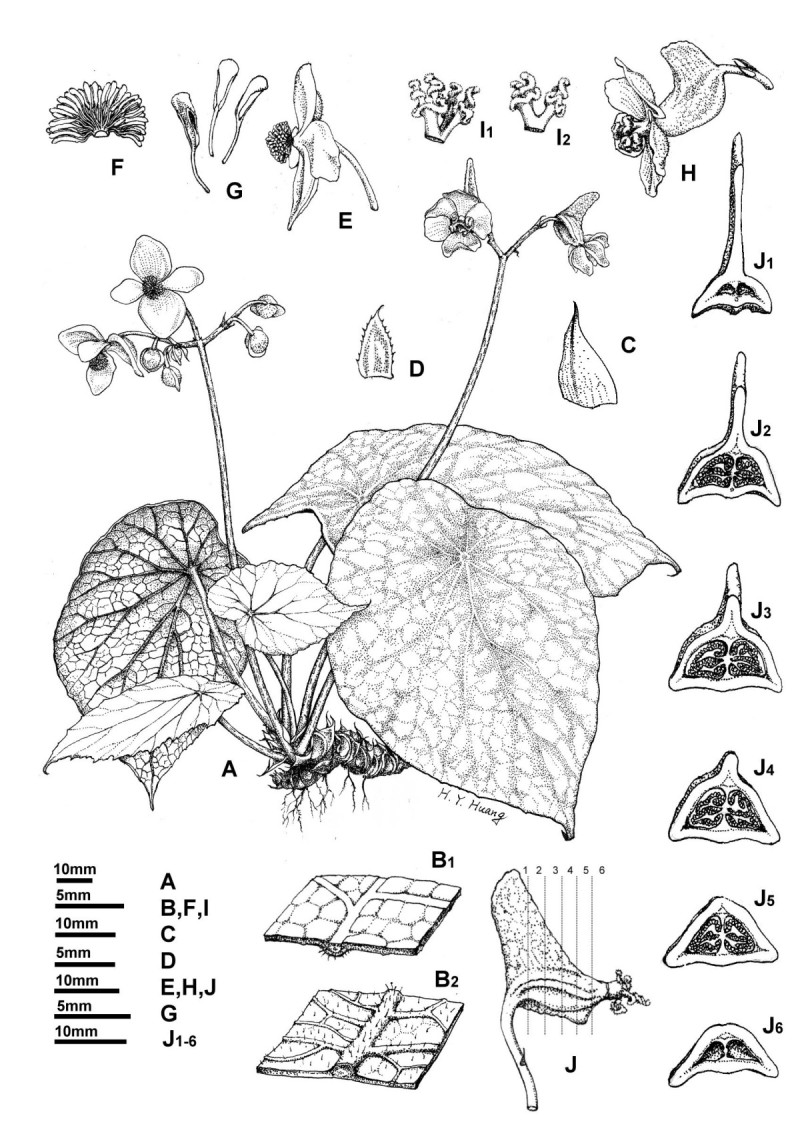
***Begonia wui-senioris*** **C.-I Peng. A**, Habit; **B**_**1**_, Leaf adaxial surface; **B**_**2**_, Leaf abaxial surface; **C**, Stipule; **D**, Bract; **E**, Staminate flower; **F**, Androecium; **G**, Stamens; **H**, Carpellate flower; **I**_**1**_, **I**_**2**_, Style and Stigmas; **J**, Fruit; **J**_**1-6**_, Serial cross sections of fruit. All from *C.-I Peng 22199* (HAST).

**Figure 2:**
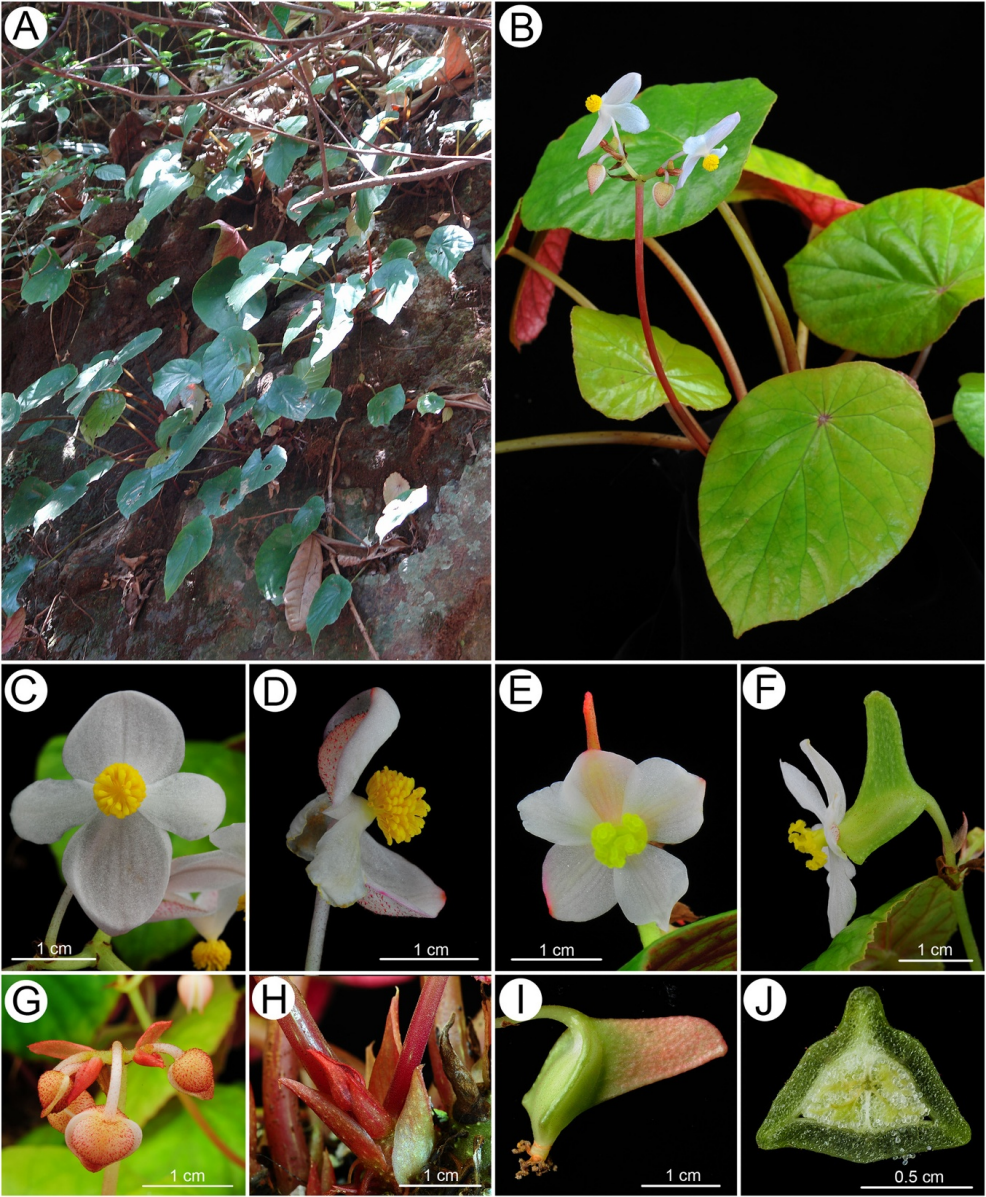
***Begonia wui-senioris*** **C.-I Peng** ***.*** **A**, Habit and habitat; **B**, Cultivated plant at anthesis; **C**, Staminate flower, face view; **D**, Staminate flower, side view; **E**, Carpellate flower, face view; **F**, Carpellate flower, side view; **G**, Inflorescence and bracts; **H**, Stipules on rhizomes; **I**, Fruit; **J**, Cross section of fruit. All from *C.-I Peng 22199* (HAST).

**Begonia wui-senioris** C.-I Peng, sp. nov. (sect. *Platycentrum*). —Type: Myanmar. Ca. 200 km northeast of Mandalay, elevation ca. 200 m, on limestone hill, 2 May 2009, specimens pressed from cultivated plants on 18 May 2011, *Ching-I Peng 22199* (holotype: HAST; isotype: E). 曼德勒秋海棠 Figures 1 and 2.

*Herbs*, monoecious, evergreen, rhizomatous. *Rhizomes* 1 cm thick, to 10 cm long, internodes ca. 0.5 cm long. *Stipules* eventually deciduous, ovate-triangular, boat-shaped, ca. 1.2 cm long, 0.9 cm wide, apex attenuate. *Leaves* peltate; petiole terete, 5–21 cm long, 0.2-0.5 cm across, green, nearly glabrous; leaf blade asymmetric, ovate, (4.5-)6.2-13(-14.8) cm long, (3-)5-8(-9.7) cm wide, apex acuminate, subcoriaceous, abaxially pale green or red, veins red and puberulous, adaxially green and glabrous. *Inflorescences* axillary, arising directly from rhizome, cymes dichasial, branched 2 or 3 times. Peduncle 6–25 cm long, 0.2 cm across, sparsely red puberulous or glabrous; bracts elliptic, 0.7 cm long, 0.3 cm wide, boat-shaped, red, sparsely ciliate, eventually deciduous. *Staminate flowers*: pedicel 1.7-2.3 cm long, red scabrous, tepals 4, outer 2 ovate, broadly ovate or suborbicular, ca. 1.4 cm long, 0.9 cm wide, white, abaxially red scabrous, inner 2 elliptic, ca. 1.2 cm long, 0.7 cm wide, white; androecium actinomorphic, spherical, ca. 0.5 cm across; stamens 60–80; anthers oblong-obovoid, apex obtuse, 2-locular. *Carpellate flower*: pedicel 1.5-2 cm long, red scabrous, tepals 5, elliptic, 1.1-1.4 cm long, 0.8-1.2 cm wide, white, abaxially red scabrous; ovary trigonous-ellipsoid, ca. 1.7 cm long, 0.3 cm thick (wings excluded), red scabrous, unequally 3-winged, 2-locular; lateral wings inconspicuous, 0.2 cm tall, greenish, abaxial wing oblong-ovate, ca. 1.7 cm tall, reddish; placentae axile, bilamellate; styles 2, fused at base, stigma spirally twisted. *Capsules* nodding, fruit body trigonous-ellipsoid, ca. 1.7 cm long, 0.3 cm thick (wings excluded), apex with persistent styles; abaxial wing trigonous to oblong-ovate, ca. 1.9 cm tall. Somatic chromosome number, 2*n* = 14 (see below).

#### Additional specimens examined

Myanmar*.* Grown at Maymyo, from plant brought from Gokteik or Kadu Hill*. J. H. Lace s. n.*, *s. d.*, (E [E00265073]); upper Central Myanmar: Mandalay Division, Peitchin Myaung Pagoda, Pyin Oo Lwin. 6 July 2001. *N. Tanaka, A. Tanaka, Than Than Aye & Khin Myo Htwe 021482* (TI).

#### Distribution

**Figure 3:**
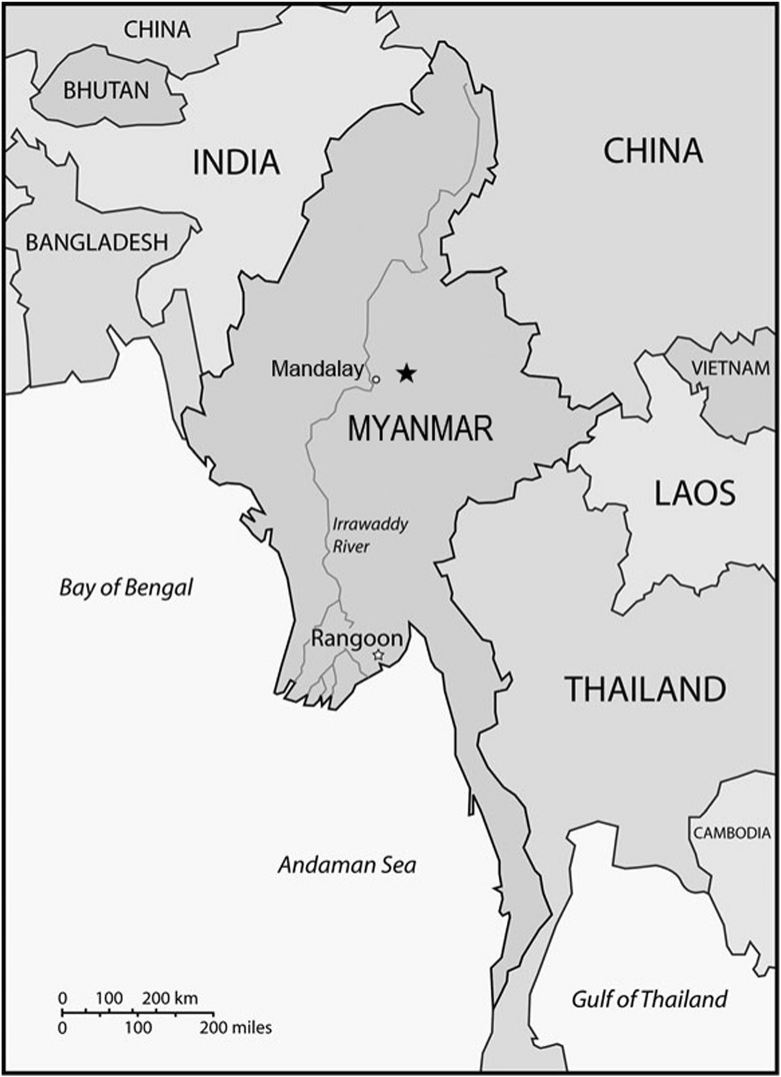
**Distribution of** ***Begonia wui-senioris*** **(★) in Myanmar.**

Known only from central Myanmar (Figure 3). John Henry Lace first collected this species in the 1910s. It was purchased by the Royal Botanic Garden Edinburgh in 1918 after Lace’s sudden death. To date, there are only three known collections of this species, all made near Mandalay.

#### Habitat

Lithophytic. Disturbed tropical rain forest; on limestone hills, on shaded, humid, rock surfaces near caves by waterfalls (based on *Peng 22199*).

#### Phenology

Flowering April to August; fruiting June to October.

#### Etymology

The specific epithet commemorates the late Prof. Zhengyi Wu, a renowned Chinese botanist.

#### Leaf anatomy

**Figure 4:**
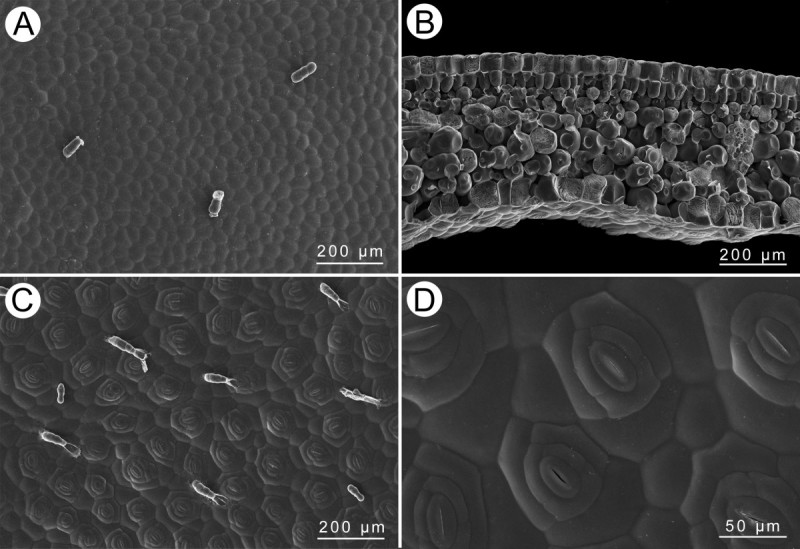
**Leaf SEM microphotographs of** ***Begonia wui-senioris*** **. A**, Adaxial surface, **B**, Cross section; **C**, Abaxial surface; **D**, Stomata complex.

Adaxial surface with scarce trichomes (Figure 4A); epidermis single-layered on both surfaces, hypodermis absent (Figure 4B); abaxial surface clothed with trichomes and very densely distributed stomata, stomata complex single, helicocytic, nearly flat (Figure 4C,D).

#### Chromosome cytology

**Figure 5:**
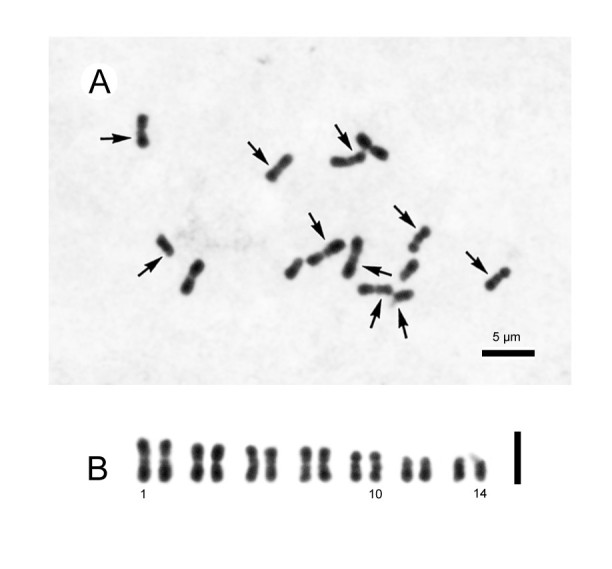
**Somatic chromosomes at metaphase of** ***Begonia wui-senioris*** **(2n = 14, from** ***C.-I Peng 22199*** **, HAST). A**, micrograph. Arrows indicate the chromosomes with secondary constrictions or satellites; **B**, somatic chromosomes serially arranged by chromosome length and centromere position. Scale bars for **A** and **B** are 5 μm.

Somatic chromosomes at metaphase of *Begonia wui-senioris* were determined to be 2*n* = 14 (Figure 5), which is the first confirmed report of the lowest chromosome number known for the genus. Somatic metaphase chromosomes of most species of *Begonia* are nearly always small (< 2 μm long), and karyotypes are mostly impossible to be determined. *Begonia wui-senioris*, however, has longer chromosomes and its karyotype is fully described here. The 14 chromosomes gradually vary in length from ca. 2.2 to 4.2 μm long. Among them, ten (Figure 5B: Nos. 1–8, 11, 12), two (Figure 5B; Nos. 9, 10) and two (Figure 5B: Nos. 13, 14) chromosomes have the centromere in the median (m), submedian (sm) and subterminal (st) positions, respectively. Most chromosomes have secondary constrictions (SC) or satellites (sat): SCs are located at interstitial regions of the long arms in four m- (Figure 5B: Nos. 1, 2, 7, 8) and two sm-chromosomes (Figure 5B: Nos. 9, 10); at interstitial region of the short arms in two m-chromosomes (Figure 5B: Nos. 5, 6); satellites were observed in the distal regions of the short arms in two st-chromosomes (Figure 5B: Nos. 13, 14). The karyotypic formula of *B. wui-senioris* is therefore 2*n* = 14 = 10m^6SC^ + 2sm^2SC^ + 2st^2sat^.

*Begonia* section *Platycentrum* in Asia comprises about 110 species (Shui et al. 2002). To our knowledge, chromosome numbers of about 30 taxa have been reported for species in this section (e.g. Ye et al. 2004; Peng et al. 2006; Li et al. 2005). The karyotypes show wide variation in number, especially in Taiwanese *Begonia* (Oginuma and Peng 2002). Among the species belonging to sect. *Platycentrum*, *B. wui-senioris* is unique in having longer chromosomes and the lowest chromosome number (2*n* = 14) for the genus. Nakata et al. (2003) documented a probable chromosome count of 2*n* = ca. 14 for a sterile, not positively identified, plant from Yunnan, China.

#### Notes

*Begonia wui-senioris, B. josephii* and *B. subperfoliata* are the only three peltate-leaved species occurring naturally in Myanmar. The latter two, however, are deciduous, tuberous species with a single leaf and a 3-locular ovary and are classified within sect. *Diploclinium*.

## Conclusion

Studies of literature and herbarium materials support the recognition of the new species, *Begonia wui-senioris*. This new species has the lowest chromosome number (2*n* = 14) known for the genus.

## Authors’ original submitted files for images

Below are the links to the authors’ original submitted files for images. Authors’ original file for figure 1 Authors’ original file for figure 2 Authors’ original file for figure 3 Authors’ original file for figure 4 Authors’ original file for figure 5
