# Supplementary figures and images for: Begonia wui-senioris (sect. Platycentrum, Begoniaceae), a new species from Myanmar
Source: Bot Stud. 2014 Feb 1;55:13. doi: 10.1186/1999-3110-55-13 (PMC5432741; doi:10.1186/1999-3110-55-13)

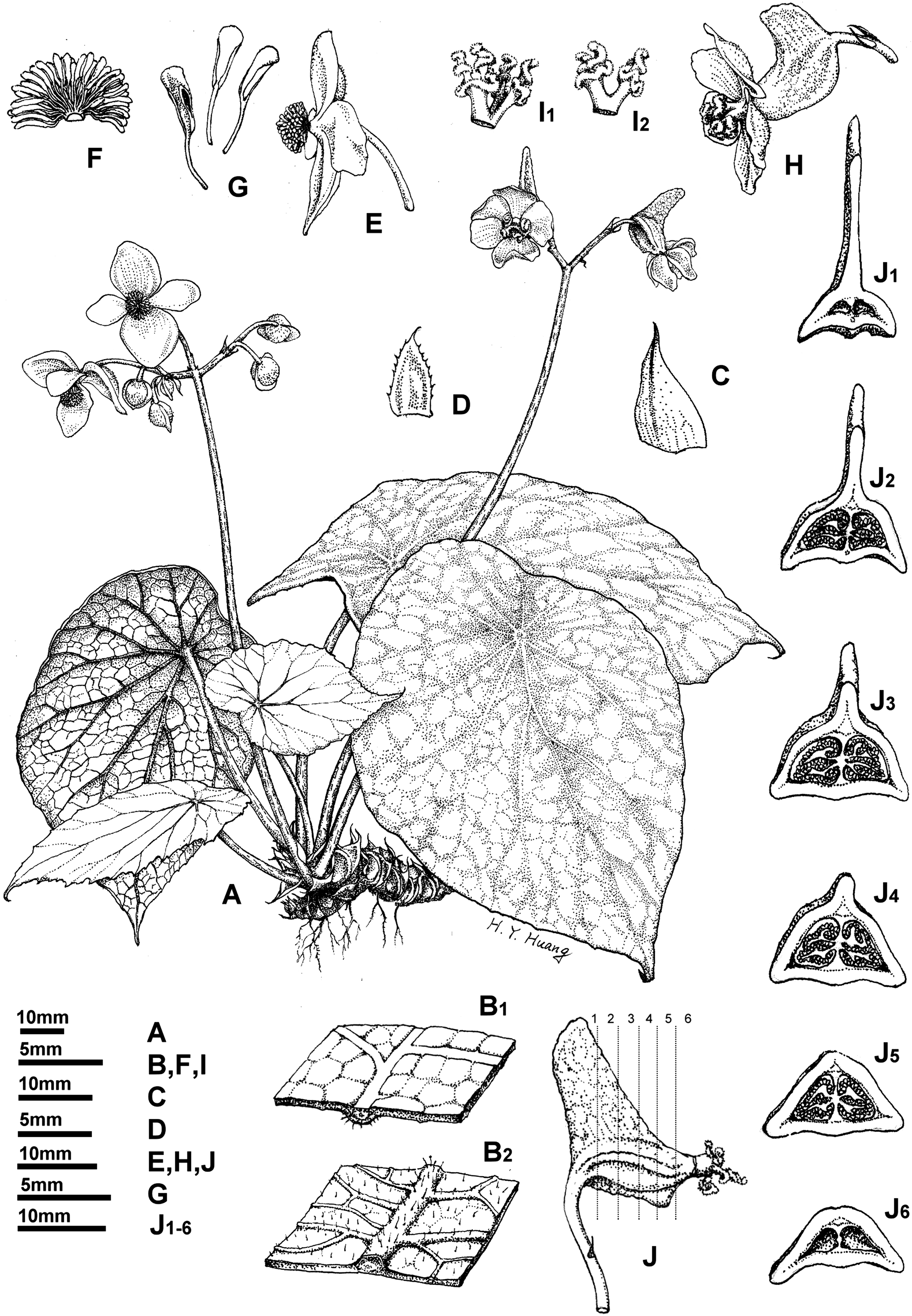

Supplement: Supplementary file 1 — Authors’ original file for figure 1 [file 40529_2013_74_MOESM1_ESM.tif]

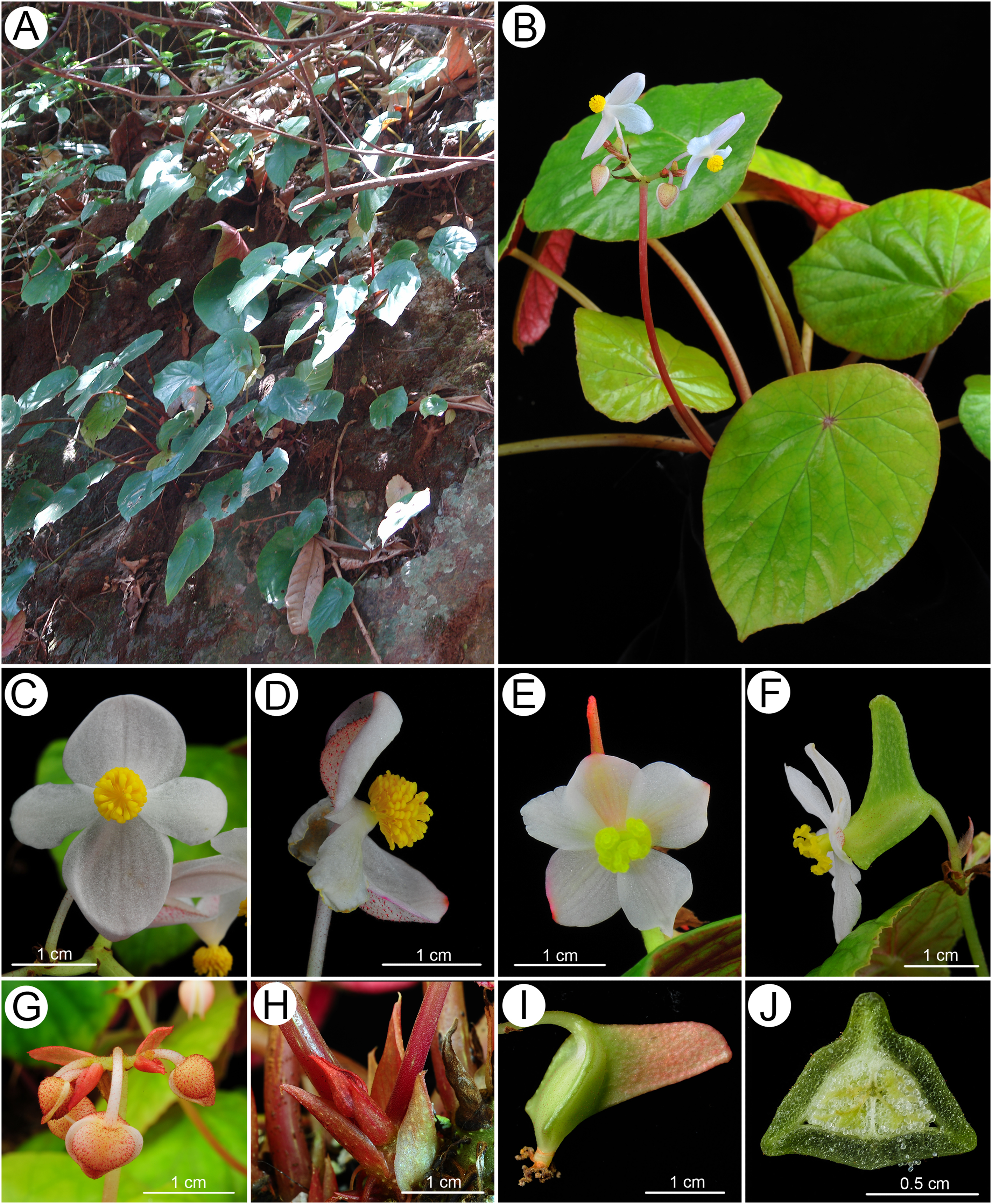

Supplement: Supplementary file 2 — Authors’ original file for figure 2 [file 40529_2013_74_MOESM2_ESM.tif]

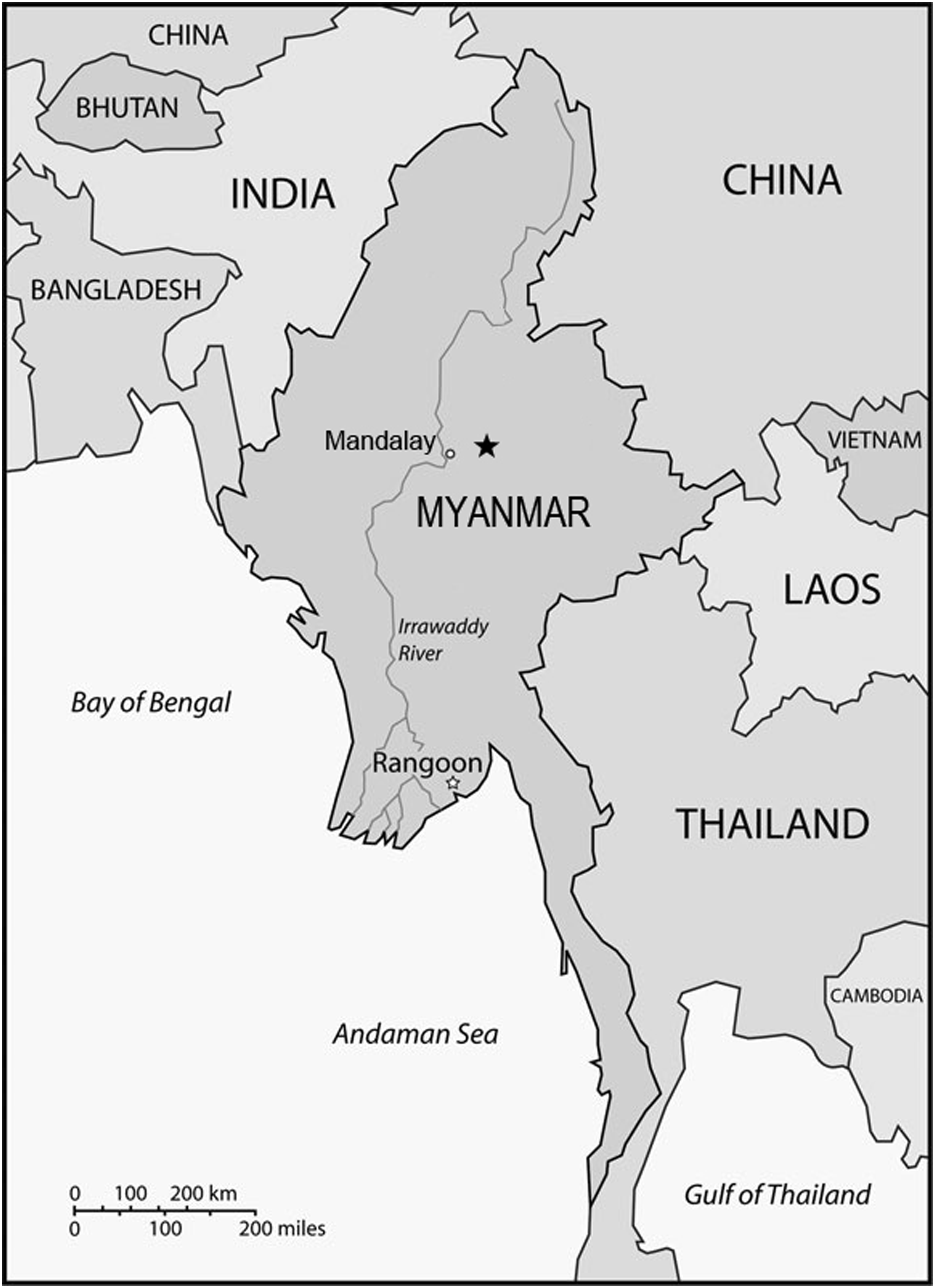

Supplement: Supplementary file 3 — Authors’ original file for figure 3 [file 40529_2013_74_MOESM3_ESM.tif]

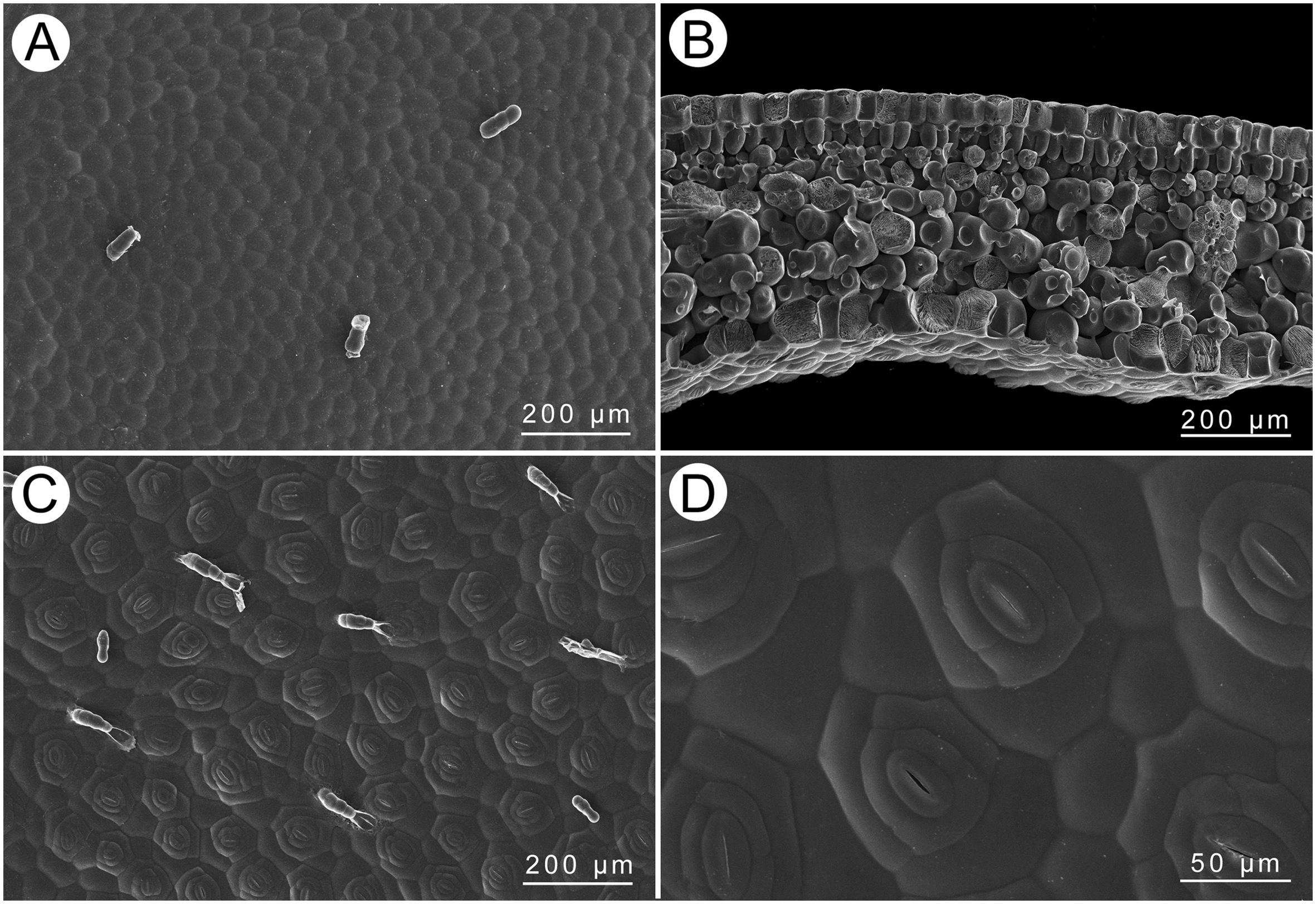

Supplement: Supplementary file 4 — Authors’ original file for figure 4 [file 40529_2013_74_MOESM4_ESM.tif]

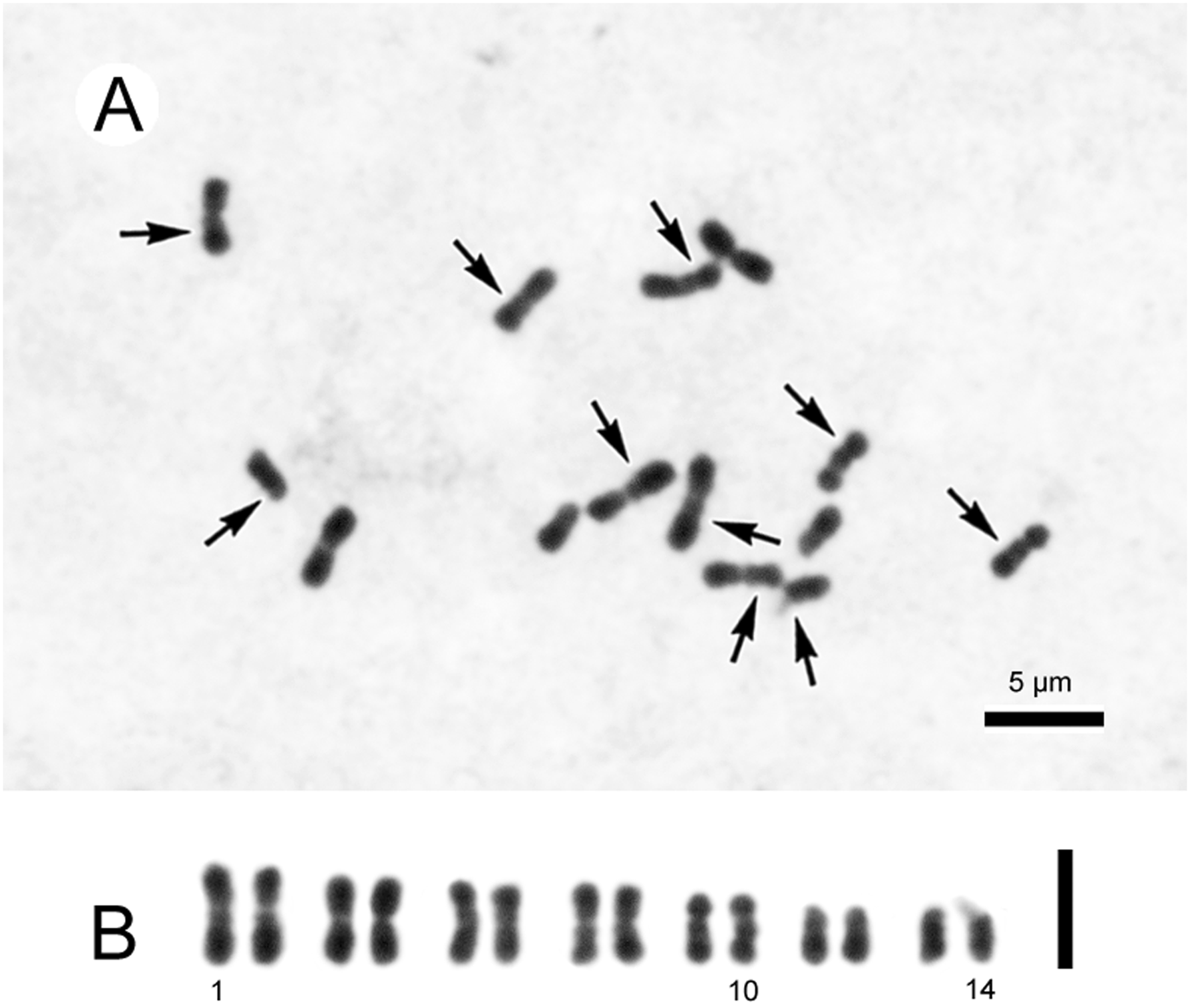

Supplement: Supplementary file 5 — Authors’ original file for figure 5 [file 40529_2013_74_MOESM5_ESM.tif]
